# Neutrophil-to-Lymphocyte Ratio as a Factor Predicting Radiotherapy Induced Oral Mucositis in Head Neck Cancer Patients Treated with Radiotherapy

**DOI:** 10.3390/jcm10194444

**Published:** 2021-09-27

**Authors:** Iwona Homa-Mlak, Anna Brzozowska, Radosław Mlak, Aneta Szudy-Szczyrek, Teresa Małecka-Massalska

**Affiliations:** 1 Department of Human Physiology, Medical University of Lublin, Radziwiłłowska 11, 20-080 Lublin, Poland; radoslaw.mlak@umlub.pl (R.M.); teresa.malecka-massalska@umlub.pl (T.M.-M.); 2 Department of Oncology, Medical University of Lublin, Jaczewskiego 7, 20-090 Lublin, Poland; annabrzozowska@umlub.pl; 3 Department of Hematooncology and Bone Marrow Transplantation, Medical University of Lublin, 20-081 Lublin, Poland; aneta.szudy-szczyrek@umlub.pl

**Keywords:** oral mucositis, survival, radiotherapy, head and neck cancer, neutrophil-to-lymphocyte ratio

## Abstract

Background: The objective of this research conducted in head and neck cancer (HNC) patients was the assessment of the relationship between neutrophil-to-lymphocyte ratio (NLR) and the incidence of severe radiotherapy (RT) induced oral mucositis (OM), as well as overall survival (OS). Methods: The study involved 207 patients in advanced stages (III–IV) of HNC. RTOG/EORTC scale was used to assess OM. The pre-treatment NLR was specified as the absolute neutrophil count divided by the absolute lymphocyte count. Results: Starting from second to seventh week of RT, we observed a significant, positive correlation between NLR values and OM grade. From the second to seventh week of RT, higher NLR values were related with significant increases (from 2- to over 24-fold) in the risk of occurrence of more severe OM (multivariate analysis confirmed its independent influence). Moreover, multivariate analysis for survival revealed that both higher TNM stage (HR = 1.84; *p* = 0.0043) and higher NLR values (HR = 1.48; *p* = 0.0395) were independent prognostic factors. Conclusion: NLR is a simple and accurate parameter that is useful in the evaluation of the risk of more severe OM, as well as an independent prognostic factor of OS in patients subjected to RT due to HNC.

## 1. Introduction

Epithelial tumours in the head and neck area (head and neck cancer, HNC) are one of the most frequent tumours, with as many as 650,000 new cases every year [1]. Despite comprehensive, highly specialized treatment of HNC patients, including surgery, radiotherapy (RT), chemotherapy (CTH), or the combination of these methods, we still achieve moderate rates of 5-year survival (from 27% up to 69%, depending on tumour location and degree of progression) [2,3]. Radical RT, often combined with chemotherapy (C-RT), leads to complications, including severe acute radiation reaction in the area of mucosa (oral mucositis, OM). OM occurs in the majority of irradiated patients (80%), which constitutes a serious problem in everyday clinical practice [4,5,6]. From the clinical point of view, severe OM is particularly important, referred to as grades 3 and 4 according to the RTOG/EORTC scale [4]. Acute toxicities due to irradiation (e.g., OM) usually relent after a few weeks after RT. Starting from few months after RT, late toxicity may develop, including xerostomia, cavities (dental caries), and trismus due to fibrosis. The typical clinical symptom of OM is inflammation. Its gradual development manifests itself by severe pain, ulceration, dysphagia, and deterioration of the quality of life (QoL) [5,6,7]. What is more, even in 88% of patients, dysgeusia, nausea, and vomiting can be observed. They may cause dehydration, limitation in food intake, and finally lead to malnutrition disorders and/or cachexia [8,9]. In such cases, enteral nutrition delivered via nasogastric or percutaneous gastrostomy tube may be implemented [10]. In some patients (even in over 8%), various complications occur, e.g., damage to the spleen, liver, gastrointestinal bleeding, fistula formation or aspiration pneumonia. Another problem is the increased risk of developing severe viral, bacterial, and fungal infections [11]. Severe OM predisposes bacteremia and the development of sepsis [12]. Additionally, the risk is increased by salivation disorders due to irradiation [13]. Some patients (16%) even require hospitalisation due to the severity of symptoms, which is associated with the need for intensive and expensive treatment [5,6,7]. Consequences (affecting up to 62% of patients) of severe OM include eating disorders, the need to administer antibiotics, or intravenous analgesia, which is further associated with admission to or a longer stay in the hospital [14]. As a result, the total cost of the treatment increases. According to Sonis et al., the cost of treating one patient may increase even by 6000 USD [15]. In about 11% patients, due to the severity of OM, there is a need for discontinuation of RT and/or CTH [5,6,7]. The methods used to reduce OM include, above all, modern RT and intensive monitoring of patients, as well as comprehensive treatment of the already developed reaction. Longer treatment time is associated with poorer local HNC control and shorter OS. It is caused by the accelerated proliferation of neoplastic cells in response to radiation damage [16]. The doubling of the number of cancer cells is shortened from 60 days to 4 days. Due to an unplanned break in irradiation, the 2 year local control rate is reduced by 0.68%. In other studies, the authors estimate that an unplanned one day break in irradiation reduces tumour control rate at least by 1% [17]. It has been observed that both short (2–8 days) and long (>8 days) unplanned irradiation breaks are associated with shorter 5-year survival by 7% and 20%, respectively [18]. Due to the high individual variability of the occurrence of OM, previously known risk factors, such as: old age, male gender, oral hygiene, total radiation dose, smoking, systemic diseases, RT technique, and combined chemoradiation are unable to precisely identify patients with a high risk of occurrence of severe OM [5,19]. From the clinical point of view, it is important to search for such a factor because the identification of patients at risk of developing severe OM would give the opportunity to individualise the treatment plan. It is also important for this factor to be widely available, cheap, and easy to interpret.

OM develops mainly as a result of the activity of proinflammatory cytokines. After the activation of ionizing radiation, reactive oxygen species (ROS) are formed and the DNA strand is damaged. ROS and DNA breakage activate the transcription nuclear factor, kappa-light-chain-enhancer of activated B cells (NF-kB), stimulate the formation of sphingomyelinase and/or ceramide synthase, as well as fibronectin breakup. As a result of the started processes, there occurs an increase in the production of proinflammatory cytokines: IL-6, IL-1B, and TNF-α; activation of macrophages and matrix metalloproteinases (MMPs); as well as the process of apoptosis and tissue damage. In the next stages of OM development, as a result of tissue damage and co-existing infections, the action of proinflammatory cytokines, macrophages, and inflammation increases [4]. One of the markers of inflammatory reaction in the body is neutrophil-to-lymphocyte ratio (NLR) [20]. In patients with elevated NLR values, high levels of pro-inflammatory cytokines and increased peri-tumour macrophage infiltration are observed [21,22]. Systemic inflammatory response involves alterations in circulating white blood cell (WBC) counts, neutrophilia, and relative lymphopenia. Neutrophils, in turn, influence the release of circulating vascular endothelial growth factors (VEGF), which stimulate tumour angiogenesis and release IL-1, IL-6, and TNF-α involved in the development of OM [4,23,24]. It was demonstrated that NLR is also a clinically simple parameter with a confirmed prognostic value in breast, pancreatic cancer, and HNC [25,26,27,28]. Furthermore, in patients with HNC, an elevated pre-treatment NLR is a prognostic marker [20]. The hypothesis of our study assumes that NLR as an exponent of the inflammatory process may be a predictor of more severe OM in HNC patients undergoing RT. Therefore, the aim of the study was to assess pre-treatment NLR in HNC patients subjected to IMRT, and to analyse the relationship between NRL and OM severity as well as overall survival (OS).

## 2. Materials and Methods

### 2.1. Patient and Clinical Data

The study design was approved by the bioethical commission at the Medical University of Lublin (KE-0254/232/2014). Prior to the study, all patients signed the informed consent form.

This retrospective study involved 207 HNC patients in advanced stages of disease: III–IV (2014–2017). Table 1 presents detailed patient characteristics and clinical data. The disease stage has been evaluated using the 7th edition of TNM classification (UICC). Based on the pathomorphological report we noted grading which reflects the degree of histological differentiation of the tumour. Alcohol consumption was evaluated using the International Statistical Classification of Diseases and Related Health Problems (ICD). It was classified as either occasional (F10.1 and F10.2) or excessive. Only patients without infection symptoms and a normal CRP value (<5 mg/L) were included in our study. To exclude patients with infection urinalysis, CRP level assessment and chest X-ray were performed. Patients who received corticosteroids therapy one month before commencement of RT were not included in the study.

### 2.2. NLR

Blood samples for routine control were collected 1 to 3 days before the commencement of RT. The study involved patients who were not diagnosed with diseases involving the immune system (including autoimmune diseases, infectious diseases). The NLR was specified as the absolute neutrophil count divided by the absolute lymphocyte count.

### 2.3. RT

Briefly, all patients were immobilized in supine position by means of a personalised thermoplastic mask. Computer tomography (CT) scan of the analysed area was performed for a planning purpose, with 3 mm slice thickness. Bolus was not used. Own institutional treatment protocol was used to define the target volumes. The protocol complies with the International Commission on Radiation Units and Measurements Reports 50 and 62.

IMRT plan was drafted for every patient using Prowess Panther version 5.20 treatment planning system (Prowes, Inc., Concord, CA, USA). The plans utilised nine fixed-gantry angle coplanar beams with step-and-shoot treatment techniques on a linear accelerator (Siemens Artiste). For all patients, 6 MV photons were used in the treatment. For the sake of obtaining approval, the treatment plans had to meet the following criteria: (1) 95% of any PTV equal or higher than the prescribed dose; (2) 99% of any PTV equal or higher than 90% of the PTV dose. Radiation doses applied to organs at risk (OAR) were within the framework of 0225 protocol from the Radiation Therapy Oncology Group (RTOG).

The clinical target volume (CTV) for patients with a history of surgical tumour resection included surgical tumour bed with a margin of 1 cm and bilateral lymph nodes. We also used an additional boost involving high risk area (positive margins, affected lymph nodes with extracapsular extension). CTV also included a PTV margin of 3 mm. The dose for CTV (60 Gy) and high risk (66 Gy) were planned.

The patients with gross lesions had their prescribed dose of radiation defined in the following manner: a total dose of 54 Gy to low-risk targets (CTV 54), 60 Gy to the entire anatomical site and the affected lymph nodes (CTV 60), and 70 Gy to gross tumour volume with 1 cm margin (CTV 70). CTV54, CTV60, and CTV70 also included PTV margins of 3 mm. The treatment was administered once a day, over 5 fractions weekly. All patients completed the course of RT with the prescribed dose of IMRT without interruption.

Neoadjuvant CTH treatment included the following PF scheme: cisplatin (100 mg/m^2^ on day 1) and 5 FU (1000 mg/m^2^ per day, continuous infusion on days 1–5) in 21-day cycles. The concurrent chemoradiation course involved the administration of cisplatin in the dose of 100 mg/m^2^ every 21 days.

### 2.4. The Assessment of OM

RTOG/EORTC scale was used to evaluate the intensity of OM. The evaluation was performed at baseline, and, subsequently, after every week of RT (every time by the same person, medical doctor). In subsequent weeks of RT, clinically relevant highest occurring grades of OM (more severe) were analysed: in week 1: 1, in week 2: 2, in weeks 3 and 4: 3, in weeks 5 and 6: 3 or 4, in week 7: 3. Moreover, during whole treatment, weeks 1 to 7: grades 3 or 4 of OM.

### 2.5. Overall Survival

Overall survival (OS) was defined as the time from the date of RT start to the date of patient death or the date of last follow-up (censored data).

### 2.6. Statistical Analysis

Statistical analysis and graph generation was performed with use of MedCalc version 15.8 (MedCalc Software, Ostend, Belgium) software. For statistical analysis, patients were divided into 2 groups in terms of NLR (< or ≥cut-off) and OM classification (described above in the Assessment of OM section), in subsequent RT weeks. A Mann–Whitney U test was used to compare NLR values according to clinical and demographic factors (non-parametric test was used due to non-normal data distribution, Supplementary Table S1). Odds ratio (OR) was calculated to assess the risk of more severe grade OM in subsequent weeks of RT (multivariate analysis logistic regression was used). Receiver operating curves (ROC) with area under the curve (AUC) were generated to predict development of more severe OM among studied subjects (Table 2). In Table 3 and Supplementary Tables S2–S9, in the multivariate analysis we included variables from the univariate analysis for the 7th week of RT (typically (and also in this study) characterised by the highest percentage of severe OM), we decided to use *p* < 0.2 as entry criterion (thus we included: grading, TNM, and concurrent C-RT). In multivariate analysis, we also included the NLR variable (however in this case, we included results from the univariate analysis of the appropriate weeks). Using the Kaplan–Meier method, the probability of (OS) depending on clinical and demographic factors as well as NLR values were evaluated. Hazard ratios with 95% confidence intervals were calculated with use of log-rank test. Multivariate survival analysis was performed with the use of the Cox logistic regression (Table 4). In all analyses, *p* values below 0.05 were considered as statistically significant.

## 3. Results

### 3.1. Patient Characteristics

The study group consisted of 92.3% men. The median age was 62 years. Squamous cell carcinoma was diagnosed in all patients. At the time of enrolment, all patients were in advanced stage of disease (III–IV). All enrolled patients were treated with RT and received a complete treatment dose (48.3% patients were treated with surgery followed by RT; 5.8% underwent neoadjuvant chemotherapy followed by RT; 32.4% patients were treated with C-RT (13.5% received RT alone and 8.7% were subjected to concurrent C-RT)). Excessive alcohol consumption was declared by 21.3% patients. Current tobacco smokers constituted a significant majority of patients (84.1%). Table 1 presents detailed patient characteristics.

### 3.2. Comparison of NLR Values According to Demographic and Clinical Factors as Well as OM Grade after Subsequent Weeks of RT

**Figure 1:**
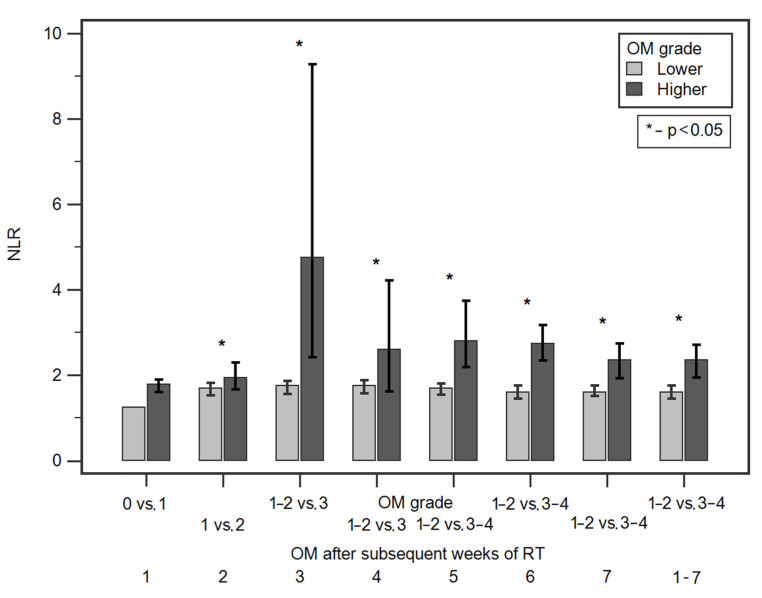
Comparisons of NLR values according to OM grade in subsequent weeks of RT.

Starting from the second week, we observed significantly higher median NLR values in patients with more severe OM grade in all subsequent weeks of RT. Table 2 and Figure 1 shows detailed data of the comparison of NLR values according to OM grade after subsequent weeks of RT. There were no statistically significant differences in NLR values according to demographic and clinical factors (Supplementary Table S1).

### 3.3. Evaluation of Diagnostic Usefulness of NLR Values in Predicting the Occurrence of More Severe OM after Subsequent Weeks of RT (ROC Analysis)

**Figure 2:**
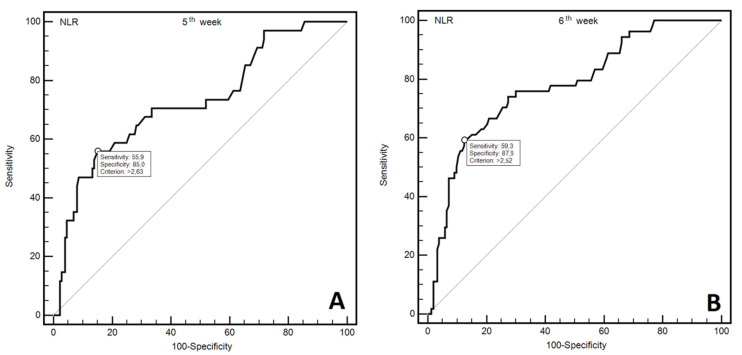
ROC curves representing diagnostic usefulness of NLR in prediction of more severe OM in 5th (**A**) and 6th (**B**) week of RT.

The analysis of ROC curves was used to assess the diagnostic utility of NLR in predicting the occurrence of severe OM (≥3 grade) in subsequent weeks of RT. We have demonstrated that NLR assessment may be helpful in predicting more severe OM following each week of RT. Statistical significance was achieved in the high diagnostic accuracy of predicting more severe OM observed, following each week of RT. Moreover, NLR was also useful in the prediction of occurrence of more severe OM (at least one episode) during all seven RT weeks. Figure 2A,B shows ROC curves for more severe OM grade for fifth and sixth week of RT. Supplementary Figure S1a–f show ROC curves for more severe OM grade for first, second, third, fourth, seventh, and 1–7 weeks of RT. Table 2 presents diagnostic usefulness of NLR values in predicting the occurrence of more severe OM after subsequent weeks of RT.

### 3.4. Assessment of the Risk of More Severe OM after Subsequent Weeks of RT According to Demographic and Clinical Factors as Well as NLR Value

NLR values exceeding 2.49 were associated with approximately a 2.5-fold increase in the risk of occurrence of more severe OM after the second week of RT (OR = 2.30; 95% CI: 2.33–4.31). This result was confirmed in multivariate analysis (OR = 2.33; 95% CI: 1.23–4.41). NLR values exceeding 2.59 were associated with approximately a 25-fold increase in the risk of occurrence of more severe OM after the third week of RT (OR = 24.66, 95% CI: 2.95–205.83). NLR values exceeding 2.49 were associated with an 8-fold increase in the risk of occurrence of more severe OM after the fourth week of RT (OR = 8.22; 95% CI: 2.10–32.24). This result was confirmed with multivariate analysis (OR = 8.45; 95% CI: 2.10–34.02). NLR values exceeding 2.63 were associated with approximately a 12.5-fold increase in the risk of occurrence of more severe OM after the fifth week of RT (OR = 12.41; 95% CI: 5.25–29.35). This result was confirmed with multivariate analysis (OR = 7.09; 95% CI: 3.17–15.76). NLR values exceeding 2.52 were associated with a 10-fold increase in the risk of occurrence of more severe OM after the sixth week of RT (OR = 10.26; 95% CI: 4.96–21.18). This result was confirmed with multivariate analysis (OR = 11.66; 95% CI: 5.45–24.96). NLR values exceeding 2.1 were associated with a 4-fold increase in the risk of occurrence of more severe OM after the seventh week of RT (OR = 3.98; 95% CI: 2.18–7.27). This result was confirmed with multivariate analysis (OR = 4.46; 95% CI: 2.38–8.33). NLR values exceeding 2.06 were associated with a 4-fold increase in the risk of occurrence of more severe OM during seven weeks of RT (OR = 4.26; 95% CI: 2.36–7.71). This result was also confirmed in multivariate analysis (OR = 4.55; 95% CI: 2.48–8.35). Table 3 shows detailed data of the assessment of the risk of more severe OM after subsequent weeks of RT according to NRL values [29].

In the first 4 weeks of RT none of the assessed demographic and clinical factors influenced the risk of occurrence of more severe OM (Supplementary Tables S2–S5). In the fifth week of RT, only tobacco smoking was related with significantly higher risk of more severe OM (OR = 37.24; 95% CI: 12.19–113.79). Similarly, in the sixth week of RT also only tobacco smoking was related with significantly higher risk of more severe OM (OR = 4.15; 95% CI: 1.21–14.20) (Supplementary Tables S6 and S7). Moreover, in the seventh week of RT, only two factors influenced a higher risk of more severe OM: both less differentiated tumours (G1 or G2) and less advanced stage of disease were related with lower risk (OR = 0.43; 95% CI: 0.24–0.78 and OR = 0.48; 95% CI: 0.26–0.91, respectively). However, in multivariate analysis, independent influence was confirmed only for TNM stage (OR = 0.63; 95% CI: 0.41–0.96). During weeks 1–7, a higher risk of more severe OM was noted in patients with less differentiated tumours (G1 or G2: OR = 0.03; 95% CI: 0.012–0.08) (Supplementary Tables S8 and S9).

### 3.5. Survival Analysis

**Figure 3:**
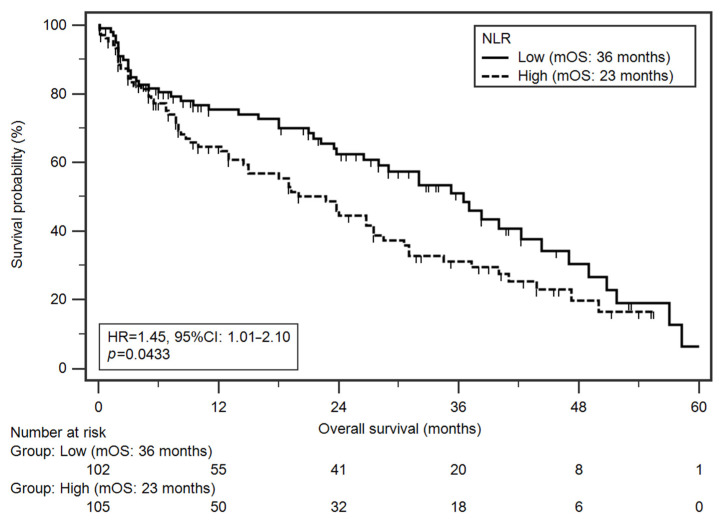
Kaplan–Meier curves illustrating death probability differences according to NLR values.

Median OS in the study group was 27 months. In total, 55.1% of patients died during follow-up. Among analysed demographic and clinical factors, a significantly higher risk of death was found in patients with more advanced disease stage (IVA–IVC vs. III, medians: 24 vs. 38 months; HR = 1.78; *p* = 0.0041) and higher NLR values (medians: 23 vs. 36 months; HR = 1.45; *p* = 0.0433; Figure 3). Multivariate analysis (results adjusted for statistically significant variables from univariate analysis) revealed that both higher TNM stage (HR = 1.84; *p* = 0.0043) and higher NLR values (HR = 1.48; *p* = 0.0395) were independent prognostic factors (Table 4) [30].

## 4. Discussion

Our study demonstrated that there is a close relationship between higher NLR values and the risk of developing severe OM in patients irradiated due to HNC. The NLR values above the specific cut-off (individual for different weeks of assessment) increased the risk of occurrence of more severe OM from 2 to approximately 25-fold, after a particular week (2 to 7) of RT.

To our knowledge, this is the first study on such a large and homogeneous (all patients received a full radiation dose and were treated with IMRT technique) group that evaluates the relationship between NLR and OM. Our research also confirmed that high NLR is an independent prognostic factor of survival.

Taken together, our results indicate that NLR is a simple and accurate parameter useful in the evaluation of the risk of the more severe OM in patients subjected to RT due to HNC. Moreover, it can be used as a prognostic factor in this group of patients.

Our study has been primarily focused on NLR as a predicting factor for OM on irradiated HNC patients. The hypothesis of our study assumed that NLR as an exponent of the inflammatory process may also be a predictor of OM, which develops on the basis of processes associated with factors typical of inflammation: IL-6, IL-1B, TNF-α, NF-kB, MMPs [4]. Identifying predictors for the development of a more severe OM may aid the implementation of appropriate and timely preventive behaviours and treatment in these patients [31,32]. The possibility of pre-treatment identification of patients with a high risk of more severe OM would enable the introduction of appropriate changes in the applied therapy. For RT, the fractionation of the total dose could be changed. In patients with an increased risk of severe OM, conventional fractionation should be considered instead of the accelerated fractionation. Alteration of the treatment in terms of reducing the radiation volume, lowering the total dose, or modification of the C-RT scheme could increase the chance of recovery. In patients with a higher risk of more severe OM, preventive actions such as adequate nutrition, supportive treatment (properly selected antibiotic therapy, anti-inflammatory, and analgesic therapy), and more frequent monitoring should be considered (especially at the early stages of treatment). In addition, there is a possibility of including patients in clinical trials testing drugs aimed at reducing the radiation reactions e.g., heparin, pentoxifylline, or dermatan sulphate [33,34,35].

It needs to be highlighted that the relationship between NLR and OM in HNC patients has not been evaluated so far. Only in patients with lung cancer has it been shown that there is a relationship between the value of NLR and the intensity of radiation pneumonitis. Lee et al. have shown that the pre-treatment level of NLR was higher in patients with radiological pneumonitis than in those without symptoms [31]. They also found that the value of NLR at the time of confirmation of radiological pneumonitis was predictive for further development of inflammation into its symptomatic form [31].

In our study, we have shown that severe OM occurred in patients with higher pre-treatment NLR values. A high NLR value indicates the possibly dominant role of neutrophilia in the development of OM.

The physiological function of neutrophils is very complex. They work in many mechanisms and have an impact on numerous metabolic processes [22,32,36,37,38]. In turn, the development of OM in HNC patients is based on over 14 previously described canonical pathways [39]. The same factors (TNF-α, MMPs, Il-1, and Il 6) take part in both processes, inflammation with the participation of neutrophils and OM.

Neutrophils enhance the synthesis of TNF-α, MMPs, Il-1, and Il 6. These factors, TNF-α, MMPs, Il-1, and Il 6, in turn, participate in several stages of the development of OM. In the second phase of primary damage response, as a result of their activation by ROS and NFkB, tissue damage and exacerbation of apoptosis occurs [4,22,32,36,37,38]. In the third phase of signal amplification, TNF-α additionally activates ceramide and caspase pathways, which together with the action of cytokines amplify the damage. Furthermore, in the fourth phase of ulceration, where there is a bacterial infection and loss of membrane continuity, TNF-α and interleukin one and six cause direct epithelial tissue damage [4].

High NLR values may indicate that neutrophilia can lead to over-stimulation of TNF-α, MMPs, Il-1, and Il 6 in the developing inflammatory reaction. The consequence may be an exacerbation of apoptosis and tissue damage. This would explain the results we obtained.

NLR is also a known marker of inflammation, and has a significant association with chronic conditions, such as cardiovascular, chronic obstructive pulmonary disease (COPD), kidney diseases, and different types of cancers [40,41,42]. In the study of over 1000 patients, it was also confirmed that it is a useful parameter to aid in the diagnosis of acute appendicitis [43]. In turn, patients with aphthous stomatitis have elevated NLR values (2.25 ± 1.07) in comparison to healthy people (1.85 ± 0.64), *p* = 0.004 [44].

In our study, NLR was measured before RT and reflected the level of inflammation before treatment. It cannot be ruled out that high NLR also indicates existing asymptomatic inflammatory processes or chronic diseases. Their presence before the start of RT could intensify the reaction in the oral cavity caused by ionising radiation. Another factor that may also exacerbate the inflammation before RT begins is smoking. It has been proven that tobacco smoking tends to activate epithelial cell intracellular signalling cascades that lead to inflammatory gene (IL-8 and TNFα) activation [45]. Our results indicate that in patients with high NLR values before RT, accurate diagnosis and assessment of possible inflammatory states and foci may be justified.

In the second part of the analysis, we assessed the value of NLR as a prognostic factor for the survival time of HNC patients. Patients with high pre-treatment NLR values were characterised by shorter OS.

On the basis of the NLR value, the prognosis can be assessed in patients with many cancers, including gastrointestinal tract malignancies, oesophageal, breast, and bladder cancer [16,46,47,48]. Templeton et al. conducted a meta-analysis including one hundred studies comprising 40,559 patients with many solid tumours. NLR greater than the median (cut-off > 4) was associated with a hazard ratio for OS of 1.81 (95% CI = 1.67 to 1.97; *p* < 0.001). High NRL as an unfavourable prognostic factor was observed in all disease subgroups, sites, and stages [49].

In patients with HNC, the value of NLR as a prognostic factor was confirmed by a meta-analysis of 15 studies with 5562 patients. It showed that elevated pre-treatment NLR significantly predicted poorer OS (HR 1.51; 95% CI 1032–1.73), and disease-specific survival (DSS) (HR 1.50; 95% CI 1.23–1.83) of HNC patients [20]. Cut-off values for NLR in the analysed studies ranged from 1.92–5.56 [20]. After the authors of the meta-analysis removed the cut-off values at the extreme 12.5% and 25%, the ranges of NLR were 2.17 to 3 and 2.38 to 2.79, respectively [20].

The reasons for this dependency are still unclear. This is probably due to the complexity of the development of the cancer process and the multitude of pathways involved in the inflammatory process, which is considered one of the potential mechanisms underlying the prognostic role of NLR. There is a change in the tumour microenvironment, and systemic inflammatory changes begin [50,51].

Neutrophilia inhibits the immune system by suppressing the cytolytic activity of immune cells, such as lymphocytes, activated T cells, and natural killer cells [32,52]. Neutrophils also affect the secretion of vascular endothelial growth factor, hepatocyte growth factor, IL-6, IL-8, elastases, and MMPs [11,21,22,23,24]. This neutrophil activity directly contributes to the severity of tumour-related angiogenesis, tumour growth, and metastasis [37].

It is known that immune cells can support anti-cancer treatment. It has been observed that not only CTH, but RT too can lead to haematological toxicity. What is interesting is that the lymphocytes are fragile for RT, and they decline early throughout irradiation. The lymphocyte depletion in the body contributes to increased tumour proliferation, facilitating metastasis and the progression of the disease. This is due to the fact that lymphocytes are responsible for the secretion of cytokines, which limit the proliferation and metastasis of tumour cells and a worse response to anti-cancer treatment. Lymphopenia is a negative prognostic factor in many cancers, including pancreatic cancer, non-small cell lung cancer (NSCLC), glioblastoma, and head and neck cancer [53,54]. Currently, there are a lack of effective methods to prevent the development of OM or to treat it. In clinical practice, antibiotics, antifungal drugs, analgesics, anti-inflammatory drugs, hydration, and nutrition support are used. Basic oral hygiene is recommended. Currently, many studies (at various stages) on drugs that inhibit the development of OM are ongoing. Tested substances include cytoprotective agents, anti-inflammation agents, cytokine production inhibitors, growth factors, antioxidant agents [55,56,57,58].

To date, there are no studies assessing the role of inhibitors of neutrophil cytokines in the development of OM in patients irradiated due to HNC. Our study demonstrates the potentially important role of NLR assessment in OM severity prediction, and indicates an urgent need for studies (also effectiveness and safety of this type of therapy) on the possibility of using inhibitors of neutrophil cytokine in the prevention and treatment of OM in patients irradiated due to HNC.

HNCs vary in the location of the tumour, and our study group reflects this diversity while at the same time being homogeneous in terms of the type of treatment (all patients received IMRT). In their study, Vera-Llonch et al. observed a higher risk of severe OM in patients with HNC of nasopharyngeal or oropharyngeal (OR 10.1 (95% CI: 2.1–49.9) and OR 6.9 (95% CI: 2.4–19.7), respectively) [59]. In contrast, this observation was not confirmed in our population of patients.

Although the study was conducted on a large group of 207 patients with HNC, there are several limitations to our study. NLR as a parameter reflecting the inflammatory processes in the body is affected by any inflammatory condition. First of all, the influence of surgery on the predictive value of NLR in the study subjects is unknown. It is also known that the value of NLR is influenced by diseases, such as coronary disease, metabolic syndrome, and anti-inflammatory drugs [60,61]. Moreover, we have not collected nor analysed information about the occurrence of chronic kidney disease (CKD) or chronic pulmonary obstructive disease (COPD) in our patients.

The advantages of our study were undoubtedly a large and homogeneous group of patients, and the consistency shown in the OS results with the majority of literature data [20]. When assessing the NLR value, we excluded the data of patients who underwent CTH in addition to RT from the analysis, in which leukocyte growth factors were used leading to changes in the values of neutrophils and lymphocytes. This is the first study evaluating the relationship between NLR and OM.

Aggressive oncological treatment is increasingly leading to serious complications. Symptoms of severe OM decrease the QoL of patients and lead to the discontinuation of RT. It has been shown that each day without RT decreases the tumour control rate by 1% [62]. That is why it is so important to minimise side effects by, among other things, searching for OM risk predictors.

NLR is a valuable and promising predictor of OM. Most of all, this parameter is well known and based on a cheap, widely available test, which is blood count. Confirmation of its value in the OM risk assessment would enable the introduction of greater individualisation of treatment of HNC patients.

## 5. Conclusions

In conclusion, our study demonstrated that the pre-treatment NLR may be an independent, significant predictor of OM, as well as a prognostic factor in HNC patients undergoing RT. Large prospective and validation studies are warranted to confirm the optimal NLR cut-off value and its predictive significance in this group of patients.

## Figures and Tables

**Table 1:** Characteristics of the study group.

Variable			Study Group (*n* = 207)
Gender	Male		191 (92.3%)
	Female		16 (7.7%)
Age Median (range)			62 (29–87)
Tumour location	Larynx		90 (43.5%)
	Oropharynx or hypopharynx		89 (43%)
	Oral cavity		28 (13.5%)
Histopathological diagnosis	Squamous cell carcinoma		207 (100%)
Grading	G1		39 (18.8%)
	G2		62 (30%)
	G3		106 (51.2%)
TNM stage	III		72(34.8%)
	IVA		121 (58.4%)
	IVB		1 (0.5%)
	IVC		13 (6.3%)
Performance status	1		142 (68.6%)
	2		65 (31.4%)
Type of treatment	RT	Surgery + RT Surgry + RT-66 Gy/33 fx in 7 weeks Surgery + RT-60 Gy/30 fx in 6 weeks	100 (48.3%) 23 (%) 77 (%)
		RT alone 70 Gy/35 fx in 7 weeks	28 (13.5%)
	Induction CHTH + RT 70 Gy/35 fx in 7 weeks		12 (5.8%)
	C-RT	Surgery + C-RT Surgery + C-RT-66 Gy/33 fx in 7 weeks Surgery + C-RT-60 Gy/30 fx in 6 weeks	49 (23.7%) 18 (%) 31 (%)
		Concurrent C-RT 70 Gy/35 fx in 7 weeks	18 (8.7%)
Alcohol consumption	Yes		44 (21.3%)
	No		163 (78.7%)
Tobacco smoking (Currently)	Yes		174 (84.1%)
	No		33 (15.9%)

Abbreviations: C-RT: chemoradiotherapy, CTH: chemotherapy, NLR: neutrophil-to-lymphocyte ratio, RT: radiotherapy.

**Table 2:** Comparisons of NLR values according to OM grade, evaluation of diagnostic usefulness of NLR values in predicting the occurrence of more severe OM after subsequent weeks of RT.

RT Week	OM Grade	Comparisons		ROC Curve Analysis			
		NLR Median (Interquartile Range)	*p*	Cut-Off Value	Sensitivity	Specificity	AUC [95% CI] *p*
1	0	1.24 (1.03–1.61)	0.1029	>1.61	56.7%	100%	0.74 [0.67–0.80] 0.0012 *
	1	1.79 (0.01–34.30)					
2	1	1.69 (0.01–15.90)	0.0225 *	>2.49	37.7%	79.2%	0.59 [0.52–0.66] 0.0204 *
	2	1.94(0.66–34.30)					
3	1 or 2	1.75 (1.21–2.49)	0.0005 *	>2.59	87.5%	78.4%	0.67 [0.60–0.73] <0.0001 *
	3	4.76 (2.70–7.69)					
4	1 or 2	1.72 (0.01–34.30)	0.0159 *	>2.49	72.7%	75.5%	0.72 [0.65–0.78] 0.0041 *
	3	2.60 (1.30–8.00)					
5	1 or 2	1.70 (0.01–34.30)	<0.0001 *	>2.63	55.9%	85%	0.73 [0.66–0.79] <0.0001 *
	3 or 4	2.80 (1.02–9.60)					
6	1 or 2	1.60 (0.01–34.30)	<0.0001 *	>2.52	59.3%	87.6%	0.78 [0.72–0.83] <0.0001 *
	3 or 4	2.74 (1.11–12.40)					
7	1 or 2	1.60 (0.01–15.90)	<0.0001 *	>2.1	60.3%	72.4%	0.68 [0.61–0.74] <0.0001 *
	3	2.35 (0.80–34.30)					
1–7	0, 1 or 2 3 or 4	1.60 (1.17–2.10) 2.35 (1.39–3.31)	<0.0001 *	>2.06	59.8%	75%	

Abbreviations: AUC: area under curve, CI: confidence interval, NLR: neutrophil to lymphocyte ratio, OM: oral mucositis, ROC: receiver operating characteristic. * statistically significant result.

**Table 3:** The assessment risk of more severe OM after subsequent weeks of RT.

RT Week	OM Grade	Odds Ratio			
		NLR *		Univariate	Multivariate ^a^
		<Cut-Off (%) ^r^	≥Cut-Off (%) *p*	OR [95% CI] *p*	OR [95% CI] *p*
1	0	4 (4.3%)	-	11.74 [0.62–221.05] 0.0999	96.4 [–] 0.9943
	1	88 (95.7%)	115 (100%)		
2	1	103 (68.2%)	27 (48.2%)	2.30 [2.33–4.31] 0.0090 *	2.33 [1.23–4.41] 0.0093 *
	2	48 (31.8%)	29 (51.8%)		
3	1 or 2	155 (99.4%)	44 (86.3%)	24.66 [2.95–205.83] 0.0031 *	3.09 [1.67–5.70] 0.0003
	3	1 (0.6%)	7 (13.7%)		
4	1 or 2	148 (98%)	48 (85.7%)	8.22 [2.10–32.24] 0.0025 *	8.45 [2.10–34.02] 0.0027 *
	3	3 (2%)	8 (14.3%)		
5	1 or 2	147 (90.7%)	15 (44.1%)	12.41 [5.25–29.35] <0.0001 *	7.09 [3.17–15.76] <0.0001 *
	3 or 4	15 (9.3%)	19 (55.9%)		
6	1 or 2	134 (85.9%)	19 (37.2%)	10.26 [4.96–21.18] <0.0001 *	11.66 [5.45–24.96] <0.0001 *
	3 or 4	22 (14.1%)	32 (62.7%)		
7	1 or 2	97 (77%)	37 (45.7%)	3.98 [2.18–7.27] <0.0001 *	4.46 [2.38–8.33] <0.0001 *
	3	29 (23%)	44 (54.3%)		
1–7	0, 1 or 2 ^r^	89 (74.2%)	35 (40.2%)	4.26 [2.36–7.71] <0.0001 *	4.55 [2.48–8.35] <0.0001 *
	3 or 4	31 (25.8%)	52 (59.8%)		

* cut-off value based on results presented in Table 2. ^a^ adjusted for statistically significant results of univariate analysis (see Supplementary Tables S2–S9). ^r^ reference variable for OR. Abbreviations: NLR: neutrophil to lymphocyte ratio, OM: oral mucositis, OR: odds ratio. [–] result cannot be calculated.

**Table 4:** Overall survival analysis of HNC patients undergoing RT depending on demographic and clinical factors.

Variable	Univariate			Multivariate ^b^	
	Median (Months)	HR (95% CI)	*p*	HR (95% CI)	*p*
**Sex** Men ^r^ Women	27 49	1.65 (0.85–3.20)	0.2227	1.70 (0.75–3.87)	0.2084
**Age [years] ^b^** ≥63 ^r^ <63	24 31	1.23 (0.85–1.78)	0.2558	1.41 (0.97–2.05)	0.0749
**Tumour location** Larynx ^r^ Oral cavity, oropharynx, hypopharynx	24 31	1.13 (0.78–1.64)	0.4957	1.30 (0.89–1.89)	0.1766
**TNM stage** IVA–IVC ^r^ III	24 38	1.78 (1.22–2.59)	0.0041 *	1.84 (1.21–2.78)	0.0043 *
**Performance status** 1 ^r^ 2	32 24	0.76 (0.52–1.12)	0.1888	0.74 (0.49–1.12)	0.1604
**Alcohol consumption** Yes ^r^ No	21 28	1.33 (0.82–2.13)	0.1963	1.29 (0.82–2.00)	0.2701
**Tobacco smoking** Yes ^r^ No	27 31	1.51 (0.93–2.44)	0.1443	1.44 (0.82–2.53)	0.2074
**NLR ^c^** High (≥1.76) ^r^ Low (<1.76)	23 36	1.45 (1.01–2.10)	0.0433 *	1.48 (1.02–2.15)	0.0395 *

^b^ adjusted for statistically significant results of univariate analysis. ^c^ dichotomised according to median value. ^r^ reference variable for HR. Abbreviations: CI: confidence interval, HR: hazard ratio, NLR: neutrophil-to-lymphocyte ratio. * statistically significant result.

## Data Availability

Data available on request.

## Supplementary Materials

The following are available online at https://www.mdpi.com/article/10.3390/jcm10194444/s1, Table S1: The comparison of NLR values according to demographic and clinical factors.; Table S2: Influence of demographic and clinical factors on the risk of more severe OM after 1st week of RT; Table S3: Influence of demographic and clinical factors on the risk of more severe OM after 2nd week of RT; Table S4: Influence of demographic and clinical factors on the risk of more severe OM after 3rd week of RT; Table S5: Influence of demographic and clinical factors on the risk of more severe OM after 4th week of RT; Table S6: Influence of demographic and clinical factors on the risk of more severe OM after 5th week of RT; Table S7: Influence of demographic and clinical factors on the risk of more severe OM after 6th week of RT; Table S8: Influence of demographic and clinical factors on the risk of more severe OM after 7th week of RT; Table S9: Influence of demographic and clinical factors on the risk of more severe OM during 1–7 weeks of RT.
