# Supplementary material for: Neutrophil-to-Lymphocyte Ratio as a Factor Predicting Radiotherapy Induced Oral Mucositis in Head Neck Cancer Patients Treated with Radiotherapy
Source: J Clin Med. 2021 Sep 27;10(19):4444. doi: 10.3390/jcm10194444 (PMC8509431; doi:10.3390/jcm10194444)
Supplement: Supplementary file 1 [file jcm-10-04444-s001.zip › jcm-1358952-supplementary.pdf]

Supplementary Table S1. The comparison of NLR values according to demographic and clinical factors.

Variable		NLR<br>Median<br>(interquartile range)	*P*
**Gender**	Male	1.76<br>(0.01-34.30)	0.4165
	Female	1.69<br>(0.66-12.20)	
**Age (years)**	≥63	1.85<br>(0.01-34.30)	0.0846
	<63	1.56<br>(0.02-9.60)	
**Tumor location**	Larynx	1.79<br>(0.40-34.30)	0.8680
	Oral cavity, oropharynx, hypopharynx	1.75<br>(0.01-12.40)	
**Grading**	G1	1.60<br>(0.01-12.40)	0.5448
	G2	1.70<br>(0.05-15.90)	
	G3	1.80<br>(0.40-34.30)	
**TNM stage**	III	1.73<br>(0.02-15.90)	0.2731
	IVA	1.80<br>(0.01-34.30)	
	IVB	1.89<br>(1.89-1.89)	
	IVC	1.44<br>(0.80-2.90)	
**Performance status**	1	1.70<br>(0.01-34.30)	0.1699
	2	2.01	

			(0.05-8.00)	
**Type of treatment**	**RT scheme**	RT alone	1.62 (0.02-6.00)	0.5302
		Other	1.79 (0.01-34.30)	
	**Induction CTH+RT**	Yes	1.95 (0.13-3.82)	0.6004
		No	1.75 (0.01-34.30)	
	**Concurrent C-RT**	Yes	1.70 (0.13-34.30)	0.3447
		No	1.77 (0.01-15.9)	
**Alcohol consumption**	Yes		1.56 (0.01-34.30)	0.8492
	No		1.61 (0.45-12.20)	
**Tobacco smoking**	Yes		1.76 (0.01-34.30)	0.1556
	No		1.70 (0.05-5.59)	

Abbreviations: C-RT-chemoradiotherapy, CTH-chemotherapy, NLR- neutrophil to lymphocyte ratio, RT – radiotherapy.

Supplementary Table S2. Influence of demographic and clinical factors on the risk of more severe OM after 1st week of RT.

Variable			Grade 1 OM after 1st weekof RT			
			**No**	**Yes**	***Univariate*** [c]	***Mulitivariate***
			**n=4 (1.9%)**	**n=203 (88.1%)**	***p*** **OR [95%CI]**	***p*** **OR [95%CI]**
Gender	Male [r]		3 (1.57%)	188 (98.43%)	0.2278	0.2919
	Female		1 (6.25%)	15 (93.75%)	4.18 [0.41-42.66]	0.27 [0.02-3.08]
Age (years)	≥63 [r]		2 (2.17%)	90 (97.83%)	0.8217	0.7672
	<63		2 (1.74%)	113 (98.26%)	0.80 [0.11-5.76]	1.39 [0.16-12.14]
Tumor location	Larynx [r]		2 (1.71%)	115 (98.29%)	0.7910	0.8818
	Oral cavity, oropharynx, hypopharynx		2 (2.22%)	88 (97.78%)	1.31 [0.18-9.46]	1.17 [0.15-8.82]
Grading	G1 or G2 [r]		2 (1.98%)	99 (98.02%)	0.9611	0.2034
	G3		2 (1.89%)	104 (98.11%)	0.95 [0.13-6.89]	1.06 [0.13-8.66]
TNM stage	III [r]		2 (2.78%)	70 (97.22%)	0.5254	0.4588
	IVA-IVC		2 (1.48%)	133 (98.52%)	0.53 [0.07-3.82]	1.91 [0.35-10.52]
Performance status	1 [r]		4 (2.82%)	138 (97.18%)	0.3337	0.9954
	2		-	65 (100%)	0.23 [0.01-4.23]	60.1 [-]
Type of treatment	RT scheme	Alone [r]	1 (3.45%)	27 (96.55%)	0.5083	0.8076
		Other	3 (1.68%)	176 (98.32%)	0.46 [0.05-4.57]	0.72 [0.05-10.22]
	Induction CTH+RT	Yes [r]	-	29 (100%)	0.7802	0.9981
		No	4 (2.25%)	174 (97.75%)	1.52 [0.08-29.00]	32.80 [-]
	Concurrent C-RT	Yes [r]	3 (4.35%)	66 (95.65%)	0.1162	0.9525
		No	1 (0.72%)	137 (99.28%)	0.16 [0.02-1.57]	1.07 [0.10-11.96]
Alcohol consumption	Yes [r]		1 (2.27%)	43 (97.73%)	0.8536	0.7140
	No		3 (1.84%)	160 (98.16%)	0.81 [0.08-7.95]	0.63 [0.05-7.27]
Tobacco smoking	Yes [r]		3 (1.72%)	171 (98.28%)	0.6219	0.7460
	No		1 (3.03%)	32 (96.97%)	1.78 [0.18-17.69]	1.49 [0.13-16.40]

[c] – Due to lack of statistical significance in univariate analysis for all assessed factors multivariate analysis was not performed.

[r] -refecence variable for OR

Abbreviations: CI-confidence interval, C-RT-chemoradiotherapy, CTH-chemotherapy, OM-oral mucositis, OR-odds ratio, RT-radiotherapy.

Supplementary Table S3. Influence of demographic and clinical factors on the risk of more severe OM after 2-nd week of RT.

Variable			Grade 3 OM during 2nd week of RT			
			**No**	**Yes**	***Univariate*[c]**	***Mulitivariate***
			**n=130 (62.8%)**	**n=77 (37.2%)**	***p*** **OR [95%CI]**	***p*** **OR [95%CI]**
Gender	Male[r]		119 (62.30%)	72 (37.70%)	0.6093 1.33 [0.443.98]	0.5770 0.73 [0.24-2.22]
	Female		11 (68.75%)	5 (31.25%)		
Age (years)	≥63 [r]		55 (59.78%)	37 (40.22%)	0.4218 1.26 [0.72-2.22]	0.4494 1.27 [0.69-2.35]
	<63		75 (65.22%)	40 (34.78%)		
Tumor location	Larynx [r]		72 (63.54%)	45 (38.46%)	0.9580 1.01 [0.57-1.81]	0.4909 1.23 [0.68-2.21]
	Oral cavity, oropharynx, hypopharynx		52 (61.90%)	32 (38.10%)		
Grading	G1 or G2 [r]		65 (64.36%)	36 (35.64%)	0.6516 0.88 [0.50-1.54]	0.5711 1.19 [0.65-2.16]
	G3		65 (61.32%)	41 (38.68%)		
TNM stage	III [r]		41 (56.94%)	31 (43.06%)	0.2038 1.46 [0.81-2.63]	0.0894 0.68 [0.43-1.06]
	IVA-IVC		89 (65.93%)	46 (34.07%)		
Performance status	1 [r]		89 (62.68%)	53 (37.32%)	0.9558 1.02 [0.55-1.86]	0.5710 0.83 [0.44-1.57]
	2		41 (63.08%)	24 (36.92%)		
Type of treatment	RT scheme	Alone [r]	17 (60.71%)	11 (39.29%)	0.8059 1.11 [0.49-2.51]	0.9740 1.01 [0.42-2.43]
		Other	113 (63.13%)	66 (36.87%)		
	Induction CTH+RT	Yes [r]	8 (66.67%)	4 (33.33%)	0.7756 0.84 [0.24-2.87]	0.9165 1.08 [0.27-4.25]
		No	122 (62.56%)	73 (37.44%)		
	Concurrent C-RT	Yes [r]	44 (65.67%)	23 (34.33%)	0.5548 0.83 [0.45-1.53]	0.6774 0.87 [0.46-1.66]
		No	86 (61.43%)	54 (38.57%)		

Alcohol consumption	Yes [r]	27 (61.36%)	17 (38.64%)	0.8240<br>1.08 [0.54-2.14]	0.5483<br>1.25 [0.60-2.57]
	No	103 (63.19%)	60 (36.81%)		
Tobacco smoking	Yes [r]	108 (62.07%)	66 (37.93%)	0.6167<br>1.22 [0.56-2.68]	0.7247<br>1.16 [0.51-2.60]
	No	22 (66.67%)	11 (33.33%)		

[c] – Due to lack of statistical significance in univariate analysis for all assessed factors multivariate analysis was not performed.

[r] - reference variable for ORAbbreviations: CI-confidence interval, C-RT-chemoradiotherapy, CTH-chemotherapy, OM-oral mucositis, OR-odds ratio, RT-radiotherapy.

Supplementary table S4. Influence of demographic and clinical factors on the risk of more severe OM after 3rd week of RT.

**Variable**			**No** n=199 (96.13%)	**Yes** n=8 (3.87%)	***Univariate***[c] *p* OR [95%CI]	***Multivariate*** *p* OR [95%CI]
Gender	Male[r]		183 (95.81%)	8 (4.19%)	0.7740	0.6178
	Female		16 (100%)	-	1.53 [0.08-27.68]	0.76 [0.25-2.67]
Age (years)	≥63[r]		87 (94.56%)	5 (5.44%)	0.3049	0.2371
	<63		112 (97.39%)	3 (2.61%)	2.15 [0.49-9.22]	0.67 [0.35-1.30]
Tumor location	Larynx[r]		112 (95.73%)	5 (4.27%)	0.7286	0.8364
	Oral cavity, oropharynx, hypopharynx		87 (96.67%)	3 (3.33%)	1.30 [0.30-5.57]	0.94 [0.51-1.71]
Grading	G1 or G2[r]		101 (100%)	-	0.0502	0.9438
	G3		98 (92.45%)	8 (7.55%)	0.06 [0.003-1.00]	0.98 [0.52-1.82]
TNM stage	III[r]		68 (94.44%)	4 (55.56%)	0.3643	0.2503
	IVA-IVC		131 (97.04%)	4 (2.96%)	1.92 [0.47-7.94]	0.79 [0.52-1.18]
Performance status	1[r]		136 (95.77%)	6 (4.23%)	0.6919	0.8086
	2		63 (96.92%)	2 (3.08%)	1.39 [0.27-7.08]	1.09 [0.56-2.12]
Type of treatment	RT scheme	Alone[r]	28 (100%)	-	0.4796	0.6953
		Other	171 (95.53%)	8 (4.47%)	0.35[0.02-6.30]	1.21 [0.46-3.15]
	Induction CTH+RT	Yes[r]	12 (100%)	-	0.9328	0.9286
		No	187 (95.90%)	8 (4.10%)	0.88[0.05-16.18]	1.06 [0.27-4.26]
	Concurrent C-RT	Yes[r]	64 (92.75%)	5 (7.25%)	0.0919	0.9644
		No	135 (97.83%)	3 (2.17%)	3.52 [0.81-15.17]	0.99 [0.51-1.91]
Alcohol consumption	Yes[r]		41 (93.18%)	3 (6.82%)	0.2644	0.7416
	No		158 (96.93%)	5 (3.07%)	2.31 [0.53-10.07]	1.14 [0.54-2.43]
Tobacco smoking	Yes[r]		167 (85.64%)	8 (14.36%)	0.4162	0.2629
	No		32 (100%)	-	3.30 [0.18-58.76]	0.60 [0.25-1.46]

[c] – Due to lack of statistical significance in univariate analysis for all assessed factors multivariate analysis was not performed.

[r] - reference variable for ORAbbreviations: CI-confidence interval, C-RT-chemoradiotherapy, CTH-chemotherapy, OM-oral mucositis, OR-odds ratio, RT-radiotherapy.

Supplementary table S5. Influence of demographic and clinical factors on the risk of more severe OM after 4th week of RT.

Variable			Grade 3 OM during 4th week of RT			
			**No**	**Yes**	***Univariate*[c]**	***Multivariate*[c]**
			**n=196 (94.7%)**	**n=11 (5.3%)**	***p*** **OR [95%CI]**	***p*** **OR [95%CI]**
Gender	Male[r]		181 (94.76%)	10 (5.24%)	0.8622	0.8440
	Female		15 (93.75%)	1 (6.25%)	0.83 [0.10-6.92]	1.25 [0.13-11.61]
Age (years)	≥63[r]		89 (96.74%)	3 (3.26%)	0.1799	0.3970
	<63		107 (92.24%)	9 (7.76%)	0.40 [0.11-1.53]	0.53 [0.12-2.30]
Tumor location	Larynx[r]		110 (94.02%)	7 (5.98%)	0.6259	0.5755
	Oral cavity, oropharynx, hypopharynx		86 (95.56%)	4 (4.44%)	1.37 [0.39-4.83]	1.45 [0.39-5.31]
Grading	G1 or G2[r]		98 (97.03%)	3 (2.97%)	0.1563	0.9352
	G3		98 (92.45%)	8 (7.55%)	0.37 [0.10-1.45]	1.06 [0.28-3.99]
TNM stage	III[r]		68 (94.44%)	4 (5.56%)	0.9099	0.3028
	IVA-IVC		128 (94.81%)	7 (5.19%)	1.08 [0.30-3.80]	1.55 [0.67-3.55]
Performance status	1[r]		134 (94.37%)	8 (5.63%)	0.7621	0.4573
	2		62 (95.38%)	3 (4.62%)	1.23 [0.32-4.81]	0.58 [0.14-2.44]
Type of treatment	RT scheme	Alone[r]	27 (96.43%)	1 (3.57%)	0.6612	0.8852
		Other	169 (94.41%)	10 (5.59%)	0.63 [0.08-5.09]	0.85 [0.09-8.09]
	Induction CTH+RT	Yes[r]	12 (100%)	-	0.7635	0.4772
		No	184 (94.36%)	11 (5.54%)	0.64 [0.03-11.53]	0.48 [0.06-3.67]
	Concurrent C-RT	Yes[r]	62 (92.54%)	5 (7.46%)	0.3463	0.6439
		No	134 (95.71%)	6 (4.29%)	1.80 [0.53-6.13]	1.35 [0.37-4.91]
Alcohol consumption	Yes[r]		39 (88.64%)	5 (11.36%)	0.0552	0.6584
	No		157 (96.32%)	6 (3.68%)	3.35 [0.97-11.56]	1.78 [0.14-23.13]
Tobacco smoking	Yes[r]		165 (94.83%)	9 (5.17%)	0.8350	0.3899
	No		31 (93.94%)	2 (6.06%)	0.84 [0.17-4.10]	0.47 [0.08-2.61]

[c] – Due to lack of statistical significance in univariate analysis for all assessed factors multivariate analysis was not performed.

[r] - reference variable for ORAbbreviations: CI-confidence interval, C-RT-chemoradiotherapy, CTH-chemotherapy, OM-oral mucositis, OR-odds ratio, RT-radiotherapy.

Supplementary table S6. Influence of demographic and clinical factors on the risk of more severe OM after 5th week of RT.

			Grade 3 or 4 OM during 5th week of RT			
**Variable**			**No** n=173 (83.6%)	**Yes** n=34 (16.4%)	***Univariate*** *p* OR [95%CI]	**Multivariate[c]** *p* OR [95%CI]
Gender	Male[r]		158 (82.72%)	33 (17.28%)	0.2770	0.2866
	Female		15 (93.75%)	1 (6.25%)	3.13[0.40-24.55]	0.31 [0.04-2.66]
Age (years)	≥63[r]		76 (82.61%)	16 (17.39%)	0.7373	0.4689
	<63		97 (84.35%)	18 (15.65%)	1.13 [0.54-2.37]	1.37 [0.58-3.21]
Tumor location	Larynx[r]		98 (93.76%)	19 (16.24%)	0.9344	0.9041
	Oral cavity, oropharynx, hypopharynx		75 (83.33%)	15 (16.67%)	0.97 [0.46-2.03]	0.95 [0.45-2.04]
Grading	G1 or G2[r]		89 (88.12%)	12 (11.88%)	0.0885	0.7210
	G3		84 (79.24%)	22 (20.76%)	0.51 [0.24-1.11]	0.86 [0.38-1.95]
TNM stage	III[r]		61 (84.72%)	11 (15.28%)	0.7450	0.8912
	IVA-IVC		112 (82.96%)	23 (17.04%)	0.88 [0.40-1.92]	1.04 [0.58-1.86]
Performance status	1[r]		117 (82.39%)	25 (17.61%)	0.4990	0.2199
	2		56 (86.15%)	9 (13.85%)	1.33 [0.58-3.04]	0.56 [0.23-1.40]
Type of treatment	RT scheme	Alone[r]	26 (92.86%)	2 (7.14%)	0.1707	0.2260
		Other	147 (82.12%)	32 (17.88%)	0.35 [0.08-1.56]	0.37 [.0.07-1.84]
	Induction CTH+RT	Yes[r]	10 (83.33%)	2 (16.67%)	0.9814	0.5330
		No	163 (83.59%)	32 (16.41%)	1.02 [0.21-4.87]	0.56 [0.09-3.48]
	Concurrent	Yes[r]	53 (81.54%)	12 (18.46%)	0.5931	0.4440
	C-RT	No	120 (84.51%)	22 (15.49%)	1.23 [0.57-2.68]	1.38 [0.60-3.18]
Alcohol consumption	Yes[r]		35 (79.55%)	9 (20.45%)	0.4179	0.3859
	No		138 (84.66%)	25 (15.34%)	1.42 [0.61-3.31]	2.21 [0.37-13.23]
Tobacco smoking	Yes[r]		29 (49.15%)	30 (50.85%)	<0.0001*	0.7988
	No		144 (97.30%)	4 (2.70%)	37.24 [12.19-113.79]	0.86 [0.26-2.83]

[c] - Results adjusted by tobacco smoking and NLR (see table 3).

[r] - reference variable for ORAbbreviations: CI-confidence interval, C-RT-chemoradiotherapy, CTH-chemotherapy, OM-oral mucositis, OR-odds ratio, RT-radiotherapy.

Supplementary table S7. Influence of demographic and clinical factors on the risk of more severe OM after 6th week of RT.

Variable			Grade 3 or 4 OM during 6th week of RT			
			**No**	**Yes**	***Univariate***	**Multivariate[c]**
			**n=153 (73.9%)**	**n=54 (26.1%)**	***p*** **OR [95%CI]**	***p*** **OR [95%CI]**
Gender	Male[r]		141 (73.82%)	50 (26.18%)	0.9179	0.9621
	Female		12 (75%)	4 (25%)	1.06 [0.33-3.45]	0.97 [0.25-3.78]
Age (years)	≥63[r]		67 (72.83%)	25 (27.17%)	0.7501	0.9630
	<63		86 (74.78%)	29 (25.22%)	1.11 [0.59-2.06]	1.02 [0.48-2.16]
Tumor location	Larynx[r]		85 (72.65%)	32 (27.35%)	0.6371	0.6832
	Oral cavity, oropharynx, hypopharynx		68 (75.56%)	22 (24.44%)	1.16 [0.62-2.18]	1.14 [0.60-2.17]
Grading	G1 or G2[r]		80 (79.2%)	21 (20.8%)	0.0921	0.9078
	G3		73 (68.9%)	33 (31.1%)	0.58 [0.31-1.09]	0.96 [0.46-1.99]
TNM stage	III[r]		54 (75%)	18 (25%)	0.7948	0.3662
	IVA-IVC		99 (73.33%)	36 (26.67%)	0.92 [0.48-1.77]	1.25 [0.77-2.04]
Performance status	1[r]		107 (75.35%)	35 (24.65%)	0.4863	0.6850
	2		46 (70.77%)	19 (29.23%)	0.79 [0.41-1.53]	0.85 [0.39-1.85]
Type of treatment	RT scheme	Alone[r]	21 (75%)	7 (25%)	0.8880	0.9759
		Other	132 (73.74%)	47 (26.26%)	0.94 [0.37-2.34]	0.98 [0.33-2.95]
	Induction CTH+RT	Yes[r]	8 (66.67%)	4 (33.33%)	0.5578	0.4745
		No	145 (74.36%)	50 (25.64%)	1.45 [0.41-5.02]	1.79 [0.36-8.87]
	Concurrent	Yes[r]	52 (77.61%)	15 (22.39%)	0.4027	0.0841
	C-RT	No	101 (72.14%)	39 (27.86%)	0.75 [0.38-1.49]	0.49 [0.21-1.10]
Alcohol consumption	Yes[r]		35 (79.54%)	9 (20.45%)	0.3397	0.8477
	No		118 (72.39%)	45 (27.61%)	0.67 [0.30-1.51]	0.54 [0.14-4.91]
Tobacco smoking	Yes[r]		123 (70.69%)	51 (29.31%)	0.0235*	0.1006
	No		30 (90.91%)	3 (9.09%)	4.15 [1.21-14.20]	3.09 [0.80-11.89]

[c] - Results adjusted by tobacco smoking and NLR (see table 3).
[r] - reference variable for OR
Abbreviations: CI-confidence interval, C-RT-chemoradiotherapy, CTH-chemotherapy, OM-oral mucositis, OR-odds ratio, RT-radiotherapy.

Supplementary table S8. Influence of demographic and clinical factors on the risk of more severe OM after 7th week of RT.

Variable			Grade 3 or 4 OM after 7th weekof RT			
			No<br>n=134<br>(64.7%)	Yes<br>n=73<br>(35.3%)	*Univariate*[b]<br>*p*<br>OR [95%CI]	Multivariate[d]<br>*p*<br>OR [95%CI]
Gender	Male[r]		123 (64.40%)	68 (35.60%)	0.7267	0.9227
	Female		11 (68.75%)	5 (31.25%)	1.22[0.41-3.65]	0.94 [0.29-3.06]
Age (years)	≥63 [r]		59 (64.13%)	33 (35.87%)	0.8708	0.6418
	<63		75 (65.22%)	40 (34.78%)	1.05 [0.59-1.86]	1.17 [0.61-2.24]
Tumor location	Larynx [r]		55 (61.11%)	35 (38.89%)	0.3391	0.7409
	Oral cavity, oropharynx, hypopharynx		79 (66.95%)	38 (33.05%)	1.32 [0.74-2.35]	0.74 [0.13-4.29]
Grading	G1 or G2 [r]		75 (74.26%)	26 (25.74%)	0.0056*	0.1153
	G3		59 (55.66%)	47 (44.34%)	0.43 [0.24-0.78]	0.60 [0.32-1.13]
TNM stage	III [r]		54 (75%)	18 (25%)	0.0253*	0.63 [0.41-0.96]
	IVA-IVC		80 (59.26%)	55 (40.74%)	0.48 [0.26-0.91]	
Performance status	1 [r]		90 (63.38%)	52 (36.62%)	0.5470	0.2889
	2		44 (67.69%)	21 (32.31%)	1.21 [0.65-2.25]	0.69 [0.35-1.37]
Type of treatment	RT scheme	Alone [r]	69 (69%)	31 (31%)	0.2151	0.1978
		Other	65 (60.75%)	42 (39.25%)	0.69 [0.39-1.23]	1.85 [0.72-4.73]
	Induction CTH+RT	Yes [r]	8 (66.67%)	4 (33.33%)	0.8853	0.7451
		No	126 (64.62%)	69 (35.38%)	0.91 [0.26-3.14]	0.79 [0.19-3.32]
	Concurrent C-RT	Yes [r]	9 (50%)	9 (50%)	0.1770	0.5428
		No	125 (66.14%)	64 (33.86%)	1.95 [0.74-5.16]	0.81 [0.42-1.58]
Alcohol consumption	Yes [r]		26 (59.09%)	18 (40.91%)	0.3783	0.2701
	No		108 (66.26%)	55 (33.74%)	1.36 [0.69-2.69]	2.42 [0.50-11.67]
Tobacco smoking	Yes [r]		110 (63.22%)	64 (36.78%)	0.2972	0.6526
	No		24 (72.73%)	9 (27.28%)	1.55 [0.68-3.54]	1.22 [0.51-2.95]

[c] - Results adjusted by grading, TNM stage and NLR (see table 3).
[r] - reference variable for ORAbbreviations: CI-confidence interval, C-RT-chemoradiotherapy, CTH-chemotherapy, OM-oral mucositis, OR-odds ratio, RT-radiotherapy.

Supplementary table S9. Influence of demographic and clinical factors on the risk of more severe OM during 1-7 weeks of RT.

**Variable**			**Grade 3 or 4 OM during 1-7 weeksof RT**			
			**No**<br>**n=120 (57.97%)**	**Yes**<br>**n=87 (42.03%)**	***Univariate*[b]**<br>***p***<br>**OR [95%CI]**	**Multivariate[d]**<br>***p***<br>**OR [95%CI]**
Gender	Male[r]		109 (57.07%)	82 (42.93%)	0.3673	0.4386
	Female		11 (64.71%)	5 (35.29%)	1.66[0.55-4.95]	0.63 [0.19-2.03]
Age (years)	≥63[r]		55 (56.70%)	37 (43.30%)	0.6368	0.7010
	<63		65 (56.52%)	50 (43.48%)	0.87[0.50-1.53]	0.88 [0.47-1.66]
Tumor location	Larynx[r]		70 (59.83%)	47 (40.17%)	0.5371	0.1451
	Oral cavity, oropharynx, hypopharynx		50 (55.56%)	40 (44.44%)	0.84 [0.48-1.46]	0.64 [0.35-1.17]
Grading	G1 or G2[r]		92 (91.09%)	9 (8.91%)	<0.0001*	0.2866
	G3		28 (26.41%)	78 (73.59%)	0.03[0.012-0.08]	0.12 [0.39-1.32]
TNM stage	III[r]		46 (63.89%)	26 (36.11%)	0.2087	0.2404
	IVA-IVC		74 (52.59%)	61 (47.41%)	0.69 [0.38-1.23]	1.28 [0.85-1.92]
Performance status	1[r]		80 (56.34%)	62 (43.66%)	0.4821	0.1983
	2		40 (61.54%)	25 (38.46%)	1.24 [0.68-2.26]	0.65 [0.33-1.25]
Type of treatment	RT scheme	Alone[r]	15 (53.57%)	13 (46.43%)	0.6124	0.4983
		Other	105 (58.66%)	74 (41.34%)	1.23 [0.55-2.74]	1.37 [0.55-3.39]
	Induction CTH+RT	Yes[r]	8 (66.67%)	4 (33.33%)	0.5318	0.5460
		No	112 (57.44%)	83 (42.56%)	0.67 [0.20-2.32]	0.64 [0.16-2.67]
	Concurrent C-RT	Yes[r]	40 (59.70%)	27 (40.30%)	0.7272	0.3646
		No	80 (57.14%)	60 (42.86%)	0.90 [0.50-1.63]	0.74 [0.38-1.42]
Alcohol consumption	Yes[r]		24 (54.55%)	20 (45.45%)	0.6042	0.2542
	No		96 (58.89%)	67 (41.11%)	1.19 [0.61-2.33]	2.34 [0.51-10.09]
Tobacco smoking	Yes[r]		99 (56.57%)	76 (43.43%)	0.3420	0.5694
	No		21 (65.63%)	11 (36.38%)	1.47 [0.67-3.22]	1.27 [0.55-2.93]

[c] - Results adjusted by grading, TNM stage and NLR (see table 3).
[r] - reference variable for OR
Abbreviations: CI-confidence interval, C-RT-chemoradiotherapy, CTH-chemotherapy, OM-oral mucositis, OR-odds ratio, RT-radiotherapy.
